# Using real-world evidence to complement evidence from randomized controlled trials on oral anticoagulants for stroke prevention

**DOI:** 10.1371/journal.pmed.1004449

**Published:** 2024-08-29

**Authors:** Mark J. R. Smeets, Suzanne C. Cannegieter

**Affiliations:** 1 Department of Clinical Epidemiology, Leiden University Medical Center, Leiden, the Netherlands; 2 Department of Internal Medicine, section Thrombosis and Hemostasis, Leiden University Medical Center, Leiden, the Netherlands

## Abstract

Mark J. R. Smeets and Suzanne C. Cannegieter discuss the use of real world data to complement data generated by clinical trials of systemic anticoagulants.

We read with great interest how Powell and colleagues in this issue [1] used a trial emulation approach to obtain real-world evidence (RWE) of the effectiveness and safety of direct oral anticoagulants (DOACs) in atrial fibrillation (AF). They did so by emulating the ARISTOTLE trial of apixaban versus warfarin using real-world, routinely collected data [2].

First, a cohort of patients with AF was constructed to resemble the apixaban cohort in the ARISTOTLE trial as closely as possible [2]. Next, this arm was compared against a propensity score matched cohort of patients taking warfarin and the same outcomes as measured the ARISTOTLE trial were reported. The study results, based on prespecified benchmarking criteria, closely resembled those of the original trial and the authors conclude that the emulation approach could be used to perform subgroup analyses lacking in the original trial. Such a subgroup analysis was subsequently performed in which the warfarin arm was dichotomized according to a time in therapeutic range (TTR) of <75% and ≥75%. This analysis showed that, while apixaban was non-inferior to warfarin when the TTR was <75%, it was inferior, in terms of mortality hazard, when the TTR of warfarin was ≥75%.

Since AF is a well-known risk factor for ischemic stroke, most patients with AF have a long-term indication for treatment with anticoagulants. Historically, this has been achieved using vitamin K antagonists (VKAs) such as warfarin, but a drawback of this treatment is that patients require regular therapeutic drug monitoring to ensure and maintain adequate TTR, which is determined by measurement of the international normalized ratio (INR) [3]. As an alternative, DOACs were developed, and large randomized clinical trials (RCTs) were warranted to establish whether DOACs were sufficiently effective and safe to replace VKAs for anticoagulation in AF. Three such trials (hereinafter referred to as “the DOAC trials”) followed which all determined DOACs to be either non-inferior or even superior compared to VKAs for the primary outcome of stroke or systemic embolism [2,4,5]. Based on these results, almost all guidelines now recommend DOACs as the primary preventive drug for AF [6].

As happens with most trials, the DOAC trials were not without limitations. First, for a causal comparison, consistency in the exposure and outcome is essential [7]. While this is the case for treatment with DOACs (e.g., similar exposure levels over time within a patient), treatment with VKAs is highly variable as the effectiveness of treatment depends on the TTR [3,8]. In the trials, the TTRs in the warfarin arms were relatively low (range 54% to 64%). It remained therefore unknown how treatment with DOACs would compare to VKA treatment when higher TTRs were achieved [2,4,5]. Comparing DOACs to ranges of TTR (e.g., low, middle, and high) would have expanded the repertoire of causal comparisons, essential for decision-making in individual patients.

Second, and a limitation of RCTs more generally, is that results from RCTs do not always translate to the populations in which interventions are subsequently implemented [9]. This can be attributed to the highly selected population that is included in the RCTs and the controlled setting in which they are conducted (which also applied to the DOAC trials) [10].

Lastly, all trials were sponsored by the manufacturers of the investigated DOACs, which is inevitable on the one hand as the costs for such trials are too high for public funds, but on the other hand makes it hard to exclude a conflict of interest.

In recent years, attention has been drawn to the potential benefits of RWE using real-world data (RWD) and target-trial emulation framework(s) [11,12]. The potential of RWE is multi-fold. It can be used to provide an estimate of the effect of an intervention under real-life clinical practice and outside the heavily controlled setting of RCTs [13]. It can be used to obtain evidence on interventions where trials cannot because of, for example, ethical constraints (e.g., smoking) or feasibility issues (e.g., sample-sizes) [13]. Furthermore, it can be used to expand on the results from existing trials by providing evidence for subgroups or outcomes which were not studied in the original trials [11]. Lastly, RWE studies are generally conducted with public funds and, in the case of therapeutic drug trials, are thus unconflicted by the interest of manufacturers. However, before RWE can live up to most of these potentials, it first needs to be established that studies using RWD provide valid and reliable results [12].

While the results from the study by Powell and colleagues [1] imply that VKAs are favorable for patients with high TTR regarding mortality risk, some remarks need to be made since RWE studies are not without limitations either. Because they are non-randomized, confounding by indication is a serious concern [11]. Powell and colleagues [1] argue that by obtaining results which are consistent with the original trial the emulation method is validated. However, there can also be other explanations for finding similar results between studies, such as chance and conflicting biases which cancel each other’s effect [14]. Therefore, further (repeated) validation, as in the RCT-DUPLICATE Initiative for example, is needed [15]. Furthermore, blinding, which is another important aspect of RCTs, is impossible for studies with RWD [11]. In the study by Powell and colleagues [1], the lack of blinding likely underlies the differential switching of anticoagulants during follow-up which can be a serious source of selection bias (though sensitivity analyses suggested that this effect was limited). Nevertheless, the obtained stratified results suggest that warfarin is not inferior, and possibly superior with regard to all-cause mortality, to apixaban if the warfarin treatment is of good quality (i.e., TTR >0.75). Therefore, we strengthen the caution, advocated by guidelines, for clinicians who think about switching their AF patients from a VKA to a DOAC when they have high TTRs as there appears to be no benefit [16].

Currently, RWE often follows sometime after the large RCTs. Hence, by the time RWE emerges, changes to guidelines have already been implemented based on trial results alone. However, as demonstrated by Powell and colleagues [1], RCTs evidently do not always capture the whole picture and have inherent limitations. In an ideal world, RWE studies and RCTs should be conducted in parallel so that results from both study designs can be used to better inform regulators and policy makers. The study by Powell and colleagues [1] provides an important and informative example of how this could be achieved.
